# Stable isotope variations of daily precipitation from 2014–2018 in the central United States

**DOI:** 10.1038/sdata.2019.18

**Published:** 2019-02-19

**Authors:** Chao Tian, Lixin Wang

**Affiliations:** 1 Department of Earth Sciences, Indiana University-Purdue University Indianapolis (IUPUI), Indianapolis, IN 46202, USA

**Keywords:** Environmental sciences, Hydrology

## Abstract

Stable isotopes of hydrogen and oxygen (δ^2^H, δ^18^O and δ^17^O) serve as powerful tracers in hydrological investigations. To our knowledge, daily precipitation isotope record especially ^17^O-excess is rare in the mid-latitudes. To fill such knowledge gap, daily precipitation samples (n=446) were collected from June 2014 to May 2018 in Indianapolis, Indiana, U.S. A Triple Water Vapor Isotope Analyzer (T-WVIA) based on Off-Axis Integrated Cavity Output Spectroscopy (OA-ICOS) technique was used to concurrently measure precipitation isotopic variations (δ^2^H, δ^18^O and δ^17^O). Meanwhile, ^17^O-excess and d-excess as second-order isotopic variables were calculated to provide additional information on precipitation formation and transport mechanisms. This study presents a four-year daily precipitation isotope dataset for mid-latitudes, and makes it available to researchers around the world who may use it as a reference for site comparisons and for assessing global hydrological models.

## Background & Summary

Stable isotopes of hydrogen and oxygen (δ^2^H and δ^18^O) are widely used as natural tracers in ecohydrological and hydroclimatic studies^1–5^. In recent years, with the development of high-precision analytical methods^6,7^, ^17^O (the least natural abundant (0.038%) oxygen isotope)^8^, becomes a new tracer to probe hydrological and meteorological processes.

Stable isotopic compositions of precipitation are affected by complex meteorological and geographical factors, such as atmospheric conditions at the moisture source and precipitation site, moisture transport trajectories, altitude of condensation and latitude^5,9–11^. There are two types of mass-dependent fractionation process (i.e., equilibrium fractionation and kinetic fractionation) during the precipitation formation. The individual stable isotopes (δ^2^H, δ^18^O and δ^17^O) demonstrate different sensitivities to equilibrium and kinetic fractionation processes^12^. Two second-order isotopic variables, deuterium excess (d-excess=δ^2^H − 8×δ^18^O)^13^ and ^17^O-excess (^17^O-excess=ln (δ^17^O + 1)−0.528×ln (δ^18^O + 1))^14^, can be utilized to provide additional constraints. The d-excess is sensitive to the kinetic fractionation processes due to the elimination of the ^2^H and ^18^O co-variation during the equilibrium fractionation^9,15^. The d-excess of precipitation is influenced by both moisture source temperature and relative humidity (hereafter RH). Similar to d-excess, ^17^O-excess is also sensitive to the kinetic fractionation (e.g., evaporation and condensation in supersaturation condition)^16,17^. However, theoretically ^17^O-excess is mainly sensitive to the RH due to the canceled temperature effect on ^18^O and ^17^O^14,18,19^. ^17^O-excess therefore could serve as a new tracer to better understand hydrological and meteorological processes. ^17^O-excess in polar ice cores has been used to reconstruct past climate over glacial-interglacial cycles^12,20–22^. The evolution of ^17^O-excess reflects the different microphysical processes along the squall line and is sensitive to convective processes in African precipitation^23^. Recent studies show that the relationship between ^18^O and ^17^O can be used to differentiate drought type (e.g., synoptic drought vs. local drought)^24^ and differentiate fog and dew formations at the Namib Desert^25^. Thus far, there are few studies on precipitation ^17^O-excess in the middle latitude regions^11,26^. δ^17^O measurements with acceptable precision has been challenging because of its low natural abundance. The traditional Isotope Ratio Mass Spectrometry (IRMS) technique is one of the most widely used approaches to measure δ^17^O. However, it is complicated, expensive and time-consuming, and can only be carried out in a small number of laboratories worldwide^6,19,27^. In recent years, laser absorption spectroscopy (LAS) techniques including Cavity Ring Down Spectroscopy (CRDS) and Off-Axis Integrated Cavity Output Spectroscopy (OA-ICOS) technique have been developed for δ^17^O analysis. Based on the recent assessments, the precision of CRDS and OA-ICOS δ^17^O and ^17^O-excess measurements are lower than traditional IRMS technique, but almost comparable^6,7,11,26,28^.

The objective of this article is to provide a four-year (June 2014 to May 2018) isotope (δ^2^H, δ^18^O, δ^17^O, d-excess and ^17^O-excess) dataset of daily precipitation from Indianapolis, Indiana of the central United States (39.88°N, 86.27°W; 258 m above sea level). Influencing factors of the precipitation formation at the site is relatively complicated and caused by different water vapor sources (Continental, Pacific, Atlantic, Gulf of Mexico, and Arctic)^29–31^. We provided detailed description of the instrument operation (δ^2^H, δ^18^O and δ^17^O) using Triple Water Vapor Isotope Analyzer (T-WVIA-45-EP; Los Gatos Research Inc. (LGR), Mountain View, CA, USA) based on OA-ICOS technique. Then, detailed ^17^O-excess data filter method was described which was found to be useful to quality control the dataset as demonstrated in our recent work^26^. It is the first publicly available daily precipitation isotope dataset (δ^2^H, δ^18^O, δ^17^O, d-excess and ^17^O-excess) from the central United States, which would provide valuable information for scientists for site comparisons and assessing global hydrological models.

## Methods

### Sample collections

The sampling location is Zionsville (Indianapolis), Indiana of the central United States (39.88°N, 86.27 °W). The sampling device is placed on the ground with a diameter of ~35 cm and volume of ~6000ml. We collected 446 daily precipitation samples from June 2014 to May 2018. To reduce evaporation effects on isotopes, samples were immediately transferred from the precipitation collector to sealed glass vials (Qorpak Bottles, Fisher Scientific Co. Germany) except for those occurring after midnight. In those cases, they were collected at the earliest possible time in the morning. Snowfall samples were first melted in sealed plastic bags and then poured into the vials. All of the samples were stored at 4 °C until isotope analysis. Notably, samples containing impurities were filtered with 0.45 μm syringe filters (Cellulose Nitrate Membrane Filters, GE Healthcare Co. UK) or centrifuged (Iec Centra CL2 Centrifuge, Thermo Electron Co. USA) depending on the size of the impurities before being measured. The meteorological data during the study period were obtained from the Zionsville meteorological station (https://www.wunderground.com).

### Isotope measurements

A Triple Water Vapor Isotope Analyzer (T-WVIA-45-EP; Los Gatos Research Inc. (LGR), Mountain View, CA, USA), based on Off-Axis Integrated Cavity Output Spectroscopy (OA-ICOS) technique, was used to concurrently measured three isotopic ratios (δ^2^H, δ^18^O and δ^17^O) of water vapor. Water Vapor Isotope Standard Source (WVISS, LGR, Mountain View, CA, USA) is a vaporization device without inducing isotope fractionation during the transformation of liquid water into water vapor. Through the combined operation of the WVISS and T-WVIA instruments, ^2^H/^1^H, ^18^O/^16^O and ^17^O/^16^O ratios of all the precipitation samples were continually and simultaneously measured at IUPUI (Indiana University-Purdue University Indianapolis) Ecohydrology Lab, as described in our previous studies^28,32^. Typically a minimum of 0.5 ml sample is needed to ensure the data quality. The water isotopic ratios were expressed in δ-notation as a deviation from a reference ratio:

(1)δ=RRVSMOW−1,

where R is the atomic ratio (e.g., ^2^H/H, ^18^O/^16^O or ^17^O/^16^O) of the sample, and R_VSMOW_ is the respective isotope ratio of the international standard Vienna Standard Mean Ocean Water (hereafter VSMOW).

To achieve high precision, the following procedure was followed as described in our earlier work^28,32^. The internal temperature of WVISS was preheated to 80 ^o^C to ensure complete vaporization of the liquid sample. The process usually takes about 2 h when the ambient temperature is about 25 ^o^C. The T-WVIA was also turned on about 2 h before the measurements to ensure ideal measuring conditions with chamber temperature and gas pressure being around 50 ^o^C and 40 Torr during measurements. Pipe-heating cable was used to heat the Teflon tubing connecting the WVISS and T-WVIA to avoid condensation of water vapor.

To avoid memory effects from residual water, the WVISS nebulizer was first purged for at least 2 min, and then the “stabilize” option of the device was turned on for 2 min to expel residual air inside the vaporizing chamber. The vapor concentration was adjusted by the “dilution control” knob through controlling the flow rates of dry air and the liquid water sample. All the samples were measured under 13000 ppm with higher precision based on our previous work^26,28^. Each sample was measured for 2 min, and the data output frequency was 1 Hz, which means 120 data points were generated for each sample.

### Isotope calibration and normalization

To routine checking the instrument performance, five commercially available working standards from LGR with known isotopic composition (Table 1) were analyzed as reference waters after every five precipitation samples.

Additionally, in order to reduce inter-laboratory difference using different technique and calibration methods, all of the isotope ratios were normalized using two International Atomic Energy Agency (IAEA) standards VSMOW and Standard Light Antarctic Precipitation (SLAP) as calibration materials. “Measured” δ value with respect to VSMOW was first calculated using the formula below described by Steig *et al.*^7^:

(2)δsample/VSMOWmeasured=δsample raw−δVSMOWraw(δVSMOWraw+1),

where δ is the δ^2^H, δ^18^O or δ^17^O, and “raw” value is directly derived from the ratio of measured isotopologue abundance.

Then, normalization to the VSMOW-SLAP scale was following the procedure described in Schoenemann *et al.*^27^:

(3)δsample/VSMOW−SLAPnormalized=δsample/VSMOWmeasured(δSLAP/VSMOWassigned)(δSLAP/VSMOWmeasured),

where δ is the δ^2^H, δ^18^O or δ^17^O, and the assigned values of SLAP is showed in Table 1. Here, SLAP2 is used as the replacement water standard for SLAP, which is not significantly different from SLAP for isotope values^33^. Therefore, SLAP2 is still referred as SLAP hereafter. The two international standards (VSMOW and SLAP) were measured once during each day of the measurements.

### ^17^O-excess data processing

Significant ^17^O-excess error is influenced by small peculiarities in either δ^18^O or δ^17^O due to small order of magnitude for ^17^O-excess (per meg, i.e., 0.001‰)^20^. To minimize sources of error, two types of quality control filters were used to check each individual data point. One is regression coefficient (λ = ln (δ^17^O + 1)/ln (δ^18^O + 1)), which will be the same as mass-dependent fractionation coefficient (θ) during the isotopic fractionation processes of liquid-vapor equilibrium and in water vapor diffusion in air^2,19^. The fractionation coefficient of oxygen isotope was found to be 0.511 ± 0.005 for kinetic transport effects^2^ and 0.529±0.001 for equilibrium effects^19^. The other restriction is ^17^O-excess value. Almost all of the ^17^O-excess values of global precipitation (e.g., rainfall, snowfall, and ice) fall within the range of −100 to +100 per meg^11,17,23,34–36^. Therefore, to attain better precision of ^17^O-excess, any measurements outside the 0.506 and 0.530 range, as well as outside the observed range (−100 to +100 per meg), were removed from the analysis. The final ^17^O-excess value for every precipitation sample was given as the mean value of quality-controlled data. To check the precision of our measurements, SLAP and the five working standards from LGR as mentioned above were used to calculate the precision. Additionally, Greenland Ice Sheet Precipitation (GISP), another international standard, was also measured to check the stability of our instrument precision.

### Code Availability

No custom code was used in this work.

## Data Records

**Figure 1:**
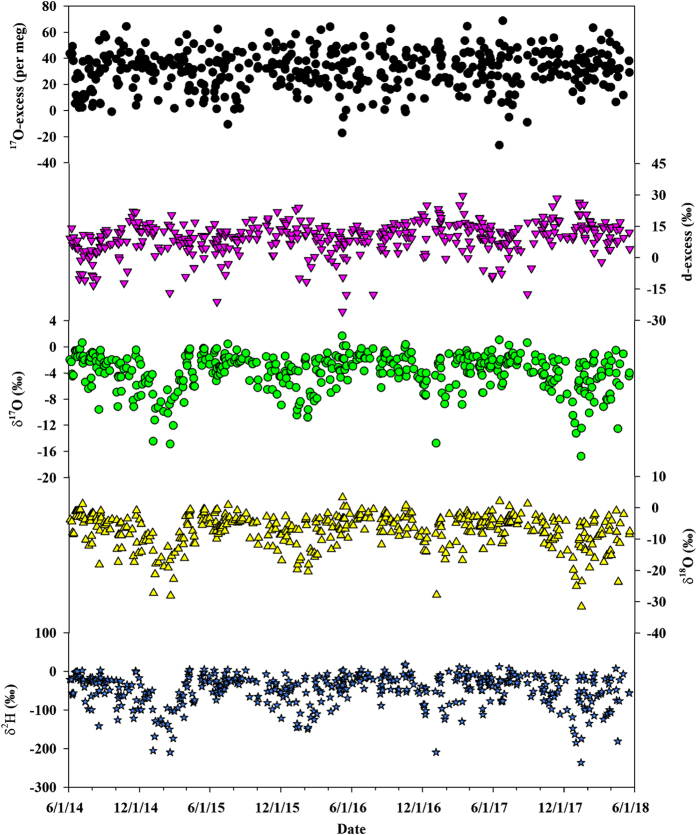
δ^2^H, δ^18^O and δ^17^O, as well as the d-excess and ^17^O-excess values of daily precipitation from June 2014 to May 2018 in Indianapolis, Indiana, U.S.

**Figure 2:**
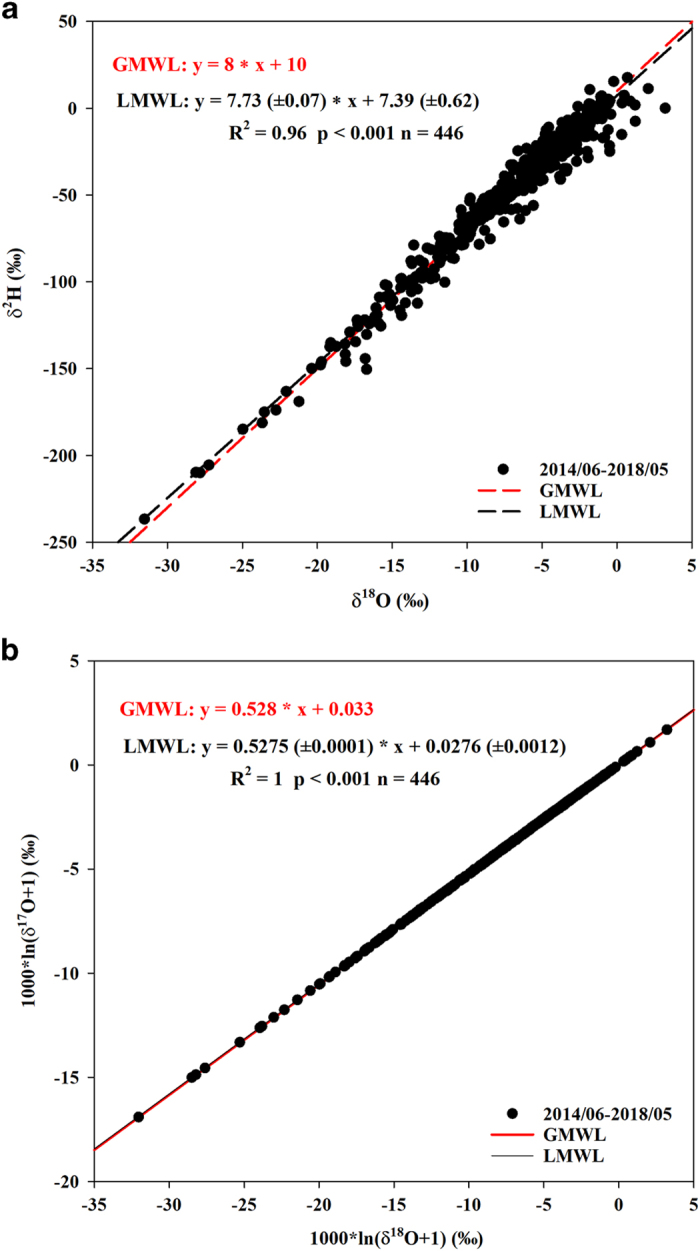
The relationships between precipitation δ^18^O and δ^2^H as well as between δ^18^O and δ^17^O during June 2014 and May 2018 in Indianapolis, Indiana, U.S. (**a**) the relationship between δ^18^O and δ^2^H; (**b**) the relationship between δ^18^O and δ^17^O.

Daily precipitation isotope database is archived in PANGAEA in a single table including 446 rows and 6 columns (Data Citation 1). Each row presents a daily precipitation event, and each column corresponds to an isotope variable including three individual stable isotopes (δ^2^H, δ^18^O and δ^17^O) and two second-order isotopic variables (d-excess and ^17^O-excess) (Table 2). Figure 1 shows a summary of the 4-year isotope record (2014 to 2018). The δ^2^H values varied from −236.75‰ to 17.64‰ with an average of −39.06‰ (Table 3). The δ^18^O values varied from −31.54‰ to 3.23‰ with an average of −6.25‰. The δ^17^O values varied from −16.77‰ to 1.68‰ with an average of −3.27‰. The d-excess values varied from −25.8‰ to 29.6‰ with an average of 9.3‰. The ^17^O-excess values varied from −26 to 69 per meg with an average of 31 per meg. The local meteoric water line (LMWL) between δ^18^O and δ^2^H based on the 446 precipitation samples in the four years was δ^2^H=7.73 (±0.07)×δ^18^O + 7.39 (±0.62) (R^2^=0.96, *p*<0.001), which is close to the Global Meteoric Water Line (GMWL, δ^2^H=8×δ^18^O + 10) (Fig. 2). The local meteoric water line (LMWL) between δ^18^O and δ^17^O was ln (δ^17^O + 1) = 0.5275 (±0.0001) × ln (δ^18^O+1) + 0.000028 (±0.000001) (R^2^=1, *p*<0.001), similar to the GMWL for oxygen (ln (δ^17^O + 1)=0.528×ln (δ^18^O + 1) + 0.000033 (R^2^=0.99999)^36^ (Fig. 2).

## Technical Validation

Multiple standards were used to validate our measurements and our measurement precision was compared with reported values in the literature (Tables 4 and 5). The precision of SLAP in our measurements was 0.79‰, 0.04‰, 0.02‰ and 3 per meg for δ^2^H, δ^18^O, δ^17^O and ^17^O-excess, respectively (Table 4). The precision of GISP was 0.12‰, 0.02‰, 0.02‰ and 7 per meg for δ^2^H, δ^18^O, δ^17^O and ^17^O-excess, respectively. The precision range for five working standards was between 0.07‰ to 0.80‰ for δ^2^H, 0.01‰ to 0.06‰ for δ^18^O, 0.02‰ to 0.03‰ for δ^17^O, and 2 to 12 per meg for ^17^O-excess.

Therefore, the ^17^O-excess precision of our OA-ICOS technique (2 to 12 per meg) is comparable with IRMS technique (0.1 to 16 per meg)^17,21,27,34–36^, as well as for CRDS method (7 to 10 per meg)^7,11^ and another type of OA-ICOS water analyzer (10 to 18 per meg)^6^ (Table 5). Meanwhile, the precisions of the three individual isotopes (δ^2^H, δ^18^O and δ^17^O) were also acceptable compared with the previous studies (Table 5).

## Additional information

**How to cite this article**: Tian, C. and Wang, L. Stable isotope variations of daily precipitation from 2014–2018 in the central United States. *Sci. Data*. 6:190018 https://doi.org/10.1038/sdata.2019.18 (2019).

**Publisher’s note**: Springer Nature remains neutral with regard to jurisdictional claims in published maps and institutional affiliations.

## Supplementary Material



## Figures and Tables

**Table 1:** The reported values of δ^2^H, δ^18^O and δ^17^O and the calculated mass-dependent fractionation coefficient (hereafter θ, θ=ln (δ^17^O+1)/ln (δ^18^O+1)), and ^17^O-excess for the five LGR working standards.

Working Standard	δ^2^H (‰)	δ^18^O (‰)	δ^17^O (‰)	θ	^17^O-excess (per meg)
LGR #1_VSMOW_	−154.0	−19.49	−10.30	0.5260	39
LGR #2_VSMOW_	−123.7	−16.24	−8.56	0.5251	48
LGR #3_VSMOW_	−97.3	−13.39	−7.06	0.5256	33
LGR #4_VSMOW_	−51.6	−7.94	−4.17	0.5242	30
LGR #5_VSMOW_	−9.2	−2.69	−1.39	0.5164	31
SLAP_VSMOW-SLAP_	−427.5^a^	−55.5^a^	−29.6986^b^	0.5280	0^b^
The reported δ^2^H and δ^18^O values from IAEA (International Atomic Energy Agency), and literature δ^17^O and ^17^O-excess values as well as the calculated θ for SLAP.					
Noting: a was from IAEA^37^; b was from Schoenemann *et al.*^27^; Los Gatos Research Inc. (LGR); Vienna Standard Mean Ocean Water (VSMOW); Standard Light Antarctic Precipitation (SLAP).					

**Table 2:** Summary of data file available.

Sample	Geographical location	Geoposition	Protocol	Data
446 daily precipitation	Indianapolis, Indiana, U.S.	39.88°N, 86.27°W; 258 m above sea level	δ^2^H, δ^18^O, δ^17^O, d-excess and ^17^O-excess	dataFile1

**Table 3:** Summary of the precipitation data record over 4 years (June 2014 to May 2018) of Indianapolis, Indiana, U.S.

	δ^2^H (‰) (Weighted)	δ^18^O (‰) (Weighted)	δ^17^O (‰) (Weighted)	d-excess (‰)	^17^O-excess (per meg)
Mean	−39.06	−6.25	−3.27	9.3	31
Standard Deviation	41.92	5.33	2.83	7.9	15
Maximum	17.64	3.23	1.68	29.6	69
Minimum	−236.75	−31.54	−16.77	−25.8	−26
Range	254.39	34.77	18.45	55.4	95

**Table 4:** Summary of the precision of δ^2^H, δ^18^O, δ^17^O and ^17^O-excess for two international standards (SLAP and GISP) and five commercially available working standards from LGR.

Samples	Precision				
	δ^2^H (‰)	δ^18^O (‰)	δ^17^O (‰)	^17^O-excess (per meg)	
SLAP_VSMOW-SLAP_	0.79	0.04	0.02	3
GISP_VSMOW-SLAP_	0.12	0.02	0.02	7
LGR #1_VSMOW-SLAP_	0.80	0.06	0.03	8
LGR #2_VSMOW-SLAP_	0.73	0.06	0.03	2
LGR #3_VSMOW-SLAP_	0.42	0.01	0.02	12
LGR #4_VSMOW-SLAP_	0.07	0.06	0.02	8
LGR #5_VSMOW-SLAP_	0.72	0.06	0.03	5
Note: Standard Light Antarctic Precipitation (SLAP); Greenland Ice Sheet Precipitation (GISP); Vienna Standard Mean Ocean Water (VSMOW); Los Gatos Research Inc. (LGR).				

**Table 5:** Summary of the precision of δ^2^H, δ^18^O, δ^17^O and ^17^O-excess from previous studies.

References	Technique	Sample type	δ^2^H (‰)	δ^18^O (‰)	δ^17^O (‰)	^17^O-excess (per meg)
Luz and Barkan *et al.*^36^^∗^	IRMS	Meteoric Water and Seawater	NA	0.02~0.03	0.02~0.03	4
Schoenemann *et al.*^27^	IRMS	GISP	NA	0.08	0.05	11
Li *et al.*^17^	IRMS	VSMOW2	NA	0.02	NA	0.1
		SLAP2	NA	0.1	NA	1
		GISP	NA	0.2	NA	11
		Vostok Antarctic Water	NA	0.3	NA	9
		West Antarctic Ice Sheet Water	NA	0.3	NA	16
Schoenemann *et al.*^21^	IRMS	West Antarctic Ice Sheet Water	NA	NA	NA	6
	CRDS	West Antarctic Ice Sheet Divide Ice Core Water	0.59	0.09	NA	NA
Steen-Larsen *et al.*^35^	IRMS	Greenland Surface Snow	NA	NA	NA	6
	CRDS	Greenland Precipitation and Surface Snow	1	0.1	NA	NA
Pang *et al.*^34^	IRMS	East Antarctic Surface Snow	0.7	0.05	0.05	5
Steig *et al.*^7^	CRDS	GISP	0.34	0.05	0.02	10
		Vostok Water	0.86	0.07	0.05	7
		West Antarctic Ice Sheet Water	0.98	0.07	0.05	10
		Kona Deep	0.32	0.02	0.02	7
Affolter *et al.*^11^	CRDS	European Precipitation and Drip Waters	0.50	0.10	0.10	10
Berman *et al.*^6^	OA-ICOS	GISP	NA	0.07	0.05	10
		USGS 45/46/47/48	NA	NA	NA	10~18
Note: ^∗^means the isotopic values are given versus VSMOW (Vienna Standard Mean Ocean Water); Isotope Ratio Mass Spectrometry (IRMS); Cavity Ring Down Spectroscopy (CRDS); Off-Axis Integrated Cavity Output Spectroscopy (OA-ICOS); Greenland Ice Sheet Precipitation (GISP); Standard Light Antarctic Precipitation (SLAP); United States Geological Survey (USGS).						
